# Infection-Induced Changes Within the Endocytic Recycling Compartment Suggest a Roadmap of Human Cytomegalovirus Egress

**DOI:** 10.3389/fmicb.2018.01888

**Published:** 2018-08-22

**Authors:** William L. Close, James E. Glassbrook, Stephen J. Gurczynski, Philip E. Pellett

**Affiliations:** ^1^Department of Microbiology, Immunology and Biochemistry, Wayne State University School of Medicine, Detroit, MI, United States; ^2^Department of Internal Medicine, University of Michigan Medical School, Ann Arbor, MI, United States

**Keywords:** human cytomegalovirus, virion envelopment and egress, virion maturation, endosecretory system, herpesvirus envelopment and egress, virus–host interactions

## Abstract

Human cytomegalovirus (HCMV) is an important pathogen in developing fetuses, neonates, and individuals with compromised immune systems. Gaps in our understanding of the mechanisms required for virion assembly stand in the way of development of antivirals targeting late stages of viral replication. During infection, HCMV causes a dramatic reorganization of the host endosecretory system, leading to the formation of the cytoplasmic virion assembly complex (cVAC), the site of virion assembly. As part of cVAC biogenesis, the composition and behavior of endosecretory organelles change. To gain more comprehensive understanding of the impact HCMV infection has on components of the cellular endocytic recycling compartment (ERC), we used previously published transcriptional and proteomic datasets to predict changes in the directionality of ERC trafficking. We identified infection-associated changes in gene expression that suggest shifts in the balance between endocytic and exocytic recycling pathways, leading to formation of a secretory trap within the cVAC. Conversely, there was a corresponding shift favoring outbound secretory vesicle trafficking, indicating a potential role in virion egress. These observations are consistent with previous studies describing sequestration of signaling molecules, such as IL-6, and the synaptic vesicle-like properties of mature HCMV virions. Our analysis enabled development of a refined model incorporating old and new information related to the behavior of the ERC during HCMV replication. While limited by the paucity of integrated systems-level data, the model provides an informed basis for development of experimentally testable hypotheses related to mechanisms involved in HCMV virion maturation and egress. Information from such experiments will provide a robust roadmap for rational development of novel antivirals for HCMV and related viruses.

## Introduction

Human cytomegalovirus (HCMV) is a high prevalence viral pathogen capable of causing severe damage and disease in developing fetuses and immunocompromised individuals. Despite its public health burden, licensed antivirals for HCMV only target viral DNA replication. This lack of antiviral target diversity has contributed to clinical emergence of antiviral-resistant strains. Increased understanding of post-nuclear events during HCMV replication could facilitate identification of druggable targets at the virus–host interface, which can then be exploited for the generation of new classes of antivirals.

In this article, we focus on the cytoplasmic stages of HCMV virion envelopment and egress, with an emphasis on how the virus manipulates the cellular endocytic recycling compartment (ERC) to enable efficient envelopment and egress of nascent virions. We do not, however, address other interesting and important aspects of virus–host interactions that connect to envelopment and egress, including the potential role of ESCRT machinery during secondary envelopment or the effects of altered cellular lipid profiles resulting from infection-induced metabolic shifts (Fraile-Ramos et al., 2007; Munger et al., 2008; Tandon et al., 2009).

**FIGURE 1:**
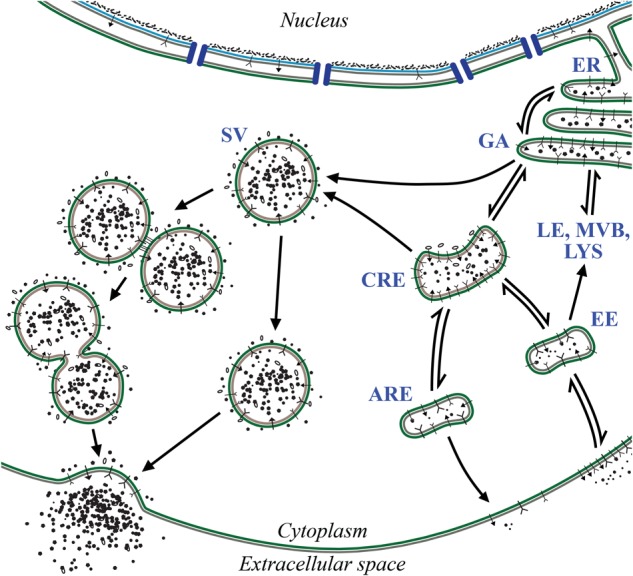
Features of the ERC. The ERC encompasses several vesicle-mediated trafficking events required for diverse cellular activities including maintenance of cellular homeostasis, regulation of growth, and response to danger. Through ERC-related pathways, membrane-bound receptors and other extracellular compounds are endocytosed and routed to the appropriate compartments for signaling, digestion, or exocytosis. Inbound materials enter through early endosome (EE) populations before being routed elsewhere such as common recycling endosomes (CRE), degradative pathways (LE, MVB, LYS), or back to the surface. From CRE compartments, cargo may also be exocytosed through apical recycling endosomes (ARE) or secretory vesicles (SV). During SV exocytosis, vesicles are transported to the plasma membrane and their contents are released by means of individual or compound exocytic events. Endoplasmic reticulum (ER), Golgi apparatus (GA), late endosomes (LE), lysosomes (LYS), multivesicular bodies (MVB).

The ERC plays a crucial role in the endocytosis and trafficking of extracellular components required for growth signaling, damage response, and maintaining homeostasis (**Figure 1**) (reviewed in Maxfield and McGraw, 2004; Rodriguez-Boulan et al., 2005; Grant and Donaldson, 2009). Trafficking through the ERC is regulated in part by membrane-bound molecules, such as Rab GTPases, which interact directly or indirectly with the cytoskeletal network. Through the ERC, materials may be endocytosed via early endosomes (EE) before being routed back along the same path and returned to the extracellular space (Thompson et al., 2007). This would be an example of the “fast” recycling pathway. Alternatively, materials may be endocytosed and transition through EE to perinuclear common recycling endosomes (CRE), which act as a hub for cytoplasmic transport, or to degradative pathways. From CRE, the cargo can be returned to the extracellular space through apical recycling endosomes (ARE), thus completing the “slow” recycling pathway, as in the case of transferrin receptor (Takahashi et al., 2012). Should materials not be recycled along either of these exocytic paths, they may be transported retrograde to the Golgi or released from the cell through secretory vesicles (SV) (Lin et al., 2004). Importantly, the forms of regulation and mechanisms of action employed along ERC pathways are highly conserved in all cell types.

To understand the role the ERC plays in HCMV replication, we begin with a brief and highly selective historical overview of our understanding of herpesvirus virion maturation and egress, a story that is parallel and intertwined with our emerging understanding of molecular and cellular biology. Helpful reviews of this history are available in early editions of Fields Virology. In depth contemporary reviews are also available as well as elsewhere in this Topic (Mettenleiter, 2006; Johnson and Baines, 2011; Pellett and Roizman, 2013; Owen et al., 2015; Cruz and Buchkovich, 2017; Close et al., 2018).

Herpesvirus virions consist of four major subassemblies: the genome, capsid, tegument, and envelope. A single molecule of the ∼236 kb double-stranded DNA genome is contained in an icosahedral capsid of *T* = 16 symmetry (Yu et al., 2017), with the capsid being surrounded by a layer of diverse proteins and RNA molecules collectively known as the tegument. This, in turn, is enclosed by a lipid bilayer envelope studded with a collection of viral glycoproteins and other membrane-associated proteins of viral and cellular origin.

Since the 1950s, ultrastructural studies that employ thin-section electron microscopy have contributed to the development of hypotheses related to the maturation of herpesvirus virions. Beginning in the 1980s, methods for manipulation of herpesvirus genomes and advances in our understanding of cell biology enabled mechanistic tests of these hypotheses. With input from many, by the early 2000s, experimental support had been obtained for the major features of the currently accepted model for herpesvirus virion maturation and egress. In this model, early assembly events occur in the nucleus, where the virus genome is replicated and packaged into newly assembled capsids (Heming et al., 2017). Viral proteins then interact with the nuclear lamina to provide mature capsids access to the inner nuclear membrane, from which the capsid acquires its primary envelope as it buds into the lumen of the nuclear membrane. This act is quickly followed by fusion of the primary envelope with the outer nuclear membrane and release of the capsid into the cytoplasm (Hellberg et al., 2016; Bigalke and Heldwein, 2017; Newcomb et al., 2017; Roller and Baines, 2017).

After capsids have emerged from the nucleus they are likely transported via microtubules through the cytoplasmic milieu (Dodding and Way, 2011), which is laden with a wide variety of macromolecules and membrane-bounded organelles. Tegument proteins form complexes between each other, capsids, and/or the cytoplasmic domains of membrane-associated viral glycoproteins. These intimate associations promote envelopment of fully tegumented capsids at membranes derived from host secretory organelles (secondary envelopment). The vesicle containing the newly enveloped virion is then transported to the plasma membrane (PM) where a membrane fusion event enables release of the nascent virion into the extracellular space. The topology of virion envelopment and egress is similar to that of exosome biogenesis and release, and involves related machinery (Sadeghipour and Mathias, 2017), which is upregulated during herpes simplex 1 infection (Birkenheuer et al., 2018). While the overall maturation and egress scheme appears to apply to all herpesviruses, details can differ from one virus to another, with the widest divergences being in the pathways employed for secondary envelopment and egress. As examples, herpes simplex virus 1 envelopment and egress is via endocytic tubules in a manner dependent on Rab5 and Rab11 (Hollinshead et al., 2012), while human herpesvirus 6B uses a CD63-linked exosomal release pathway (Mori et al., 2008).

We now turn our attention specifically to the post-nuclear maturation of HCMV. In 1988, based on studies of electron micrographs, Severi and colleagues described a model for HCMV maturation and egress that shares many important features with current models, including: (i) primary envelopment at the inner nuclear membrane followed by de-envelopment at the outer nuclear membrane, (ii) transport of naked capsids through the cytoplasm, (iii) envelopment at a cytoplasmic organelle (hypothesized to be Golgi-related), and, lastly, (iv) transport of the virion-containing vesicle to the PM for fusion-mediated virion release (Severi et al., 1988). Tooze et al. (1993) conducted pulse-chase experiments by applying horseradish peroxidase, an endocytic tracer, to HCMV-infected cells. When reacted, products of the tracer were found in what were described as tubular endosomes containing enveloped virions. Virion- and tracer-containing endosomes were not directed to late endosomes (LE) for degradation, but were released from cells, leading to the conclusion that HCMV envelopment and egress is via an early endosomal recycling compartment.

Sanchez et al. (2000a,b) published two papers that provided the first description of what we now refer to as the cytoplasmic virion assembly compartment (cVAC). They showed that the cVAC: (i) is a perinuclear compartment that overlaps with what is considered to be the ERC, (ii) lies at the center of a radially distributed microtubule network, (iii) is a structure sufficiently stable to at least partially withstand gradient purification, and (iv) is a potential site for virion assembly. Subsequent studies showed that the cVAC consists of a Golgi-derived ring that surrounds a cluster of early and recycling endosome populations and has a microtubule organizing center at its midpoint (Das et al., 2007; Das and Pellett, 2011; Das et al., 2014). During cVAC biogenesis, ERC-associated organelles undergo shifts in the relative abundances of important marker proteins that suggest transitions in their functional identities (Das and Pellett, 2011). These shifts complicate classification of host cell-derived organelles used during virion envelopment and egress (Jean Beltran et al., 2016). Indeed, typical lysosome (LYS)-associated proteins segregate into two distinct populations during HCMV infection, one, typified by LAMP1, localizes within the cVAC Golgi ring, while a population typified by LYAG localizes outside the Golgi ring. The cVAC is thus a novel, virus-induced organelle that shares machinery and many other properties with components of the ERC, but for which important spatial relationships and functional properties differ relative to uninfected cells. Based on its structure and composition, the cVAC is proposed to act at least in part as a viral assembly line that facilitates the sequential addition of the structural components required to assemble and release mature enveloped infectious virions (reviewed in Alwine, 2012; Tandon and Mocarski, 2012; Close et al., 2018).

Because biogenesis of the cVAC is dependent on viral DNA synthesis, it is apparent that viral late proteins are involved, with pUL48, pUL71, pUL94, and pUL103 having thus far been implicated in the process (Womack and Shenk, 2010; Schauflinger et al., 2011; Das et al., 2014). Of these, the tegument proteins HCMV pUL71 and pUL103 interact with each other (Fischer, 2012; Ortiz et al., 2016), as do their HSV1 homologs (Roller and Fetters, 2015; Albecka et al., 2017). This interaction may contribute to their roles in cVAC biogenesis and the similarity of the virion envelopment defects seen at late stages of the envelopment process when viruses lack either protein (Womack and Shenk, 2010; Ahlqvist and Mocarski, 2011; Schauflinger et al., 2011, 2013; Das et al., 2014). In addition, some HCMV-encoded microRNAs (miRNAs) influence organelle morphologies in ways that suggest possible roles in cVAC biogenesis or operation. For example, in uninfected cells, HCMV miRNAs can reduce expression of VAMP3, CDC42, Rab5C, Rab11A, and SNAP23 (Hook et al., 2014). While levels of these proteins are elevated in infected cells, it was to a lesser extent than in cells infected with a virus deficient in HCMV miRNAs US5-1, US5-2, and UL112-1. These viral miRNAs appear to contribute to HCMV immune evasion by trapping the inflammatory cytokines IL-6 and TNF-α in the cVAC (the cVAC secretory trap). Employing a similar immune evasion strategy, mouse cytomegalovirus (MCMV) infection leads to retention of the MHC-1 complex in an early-phase endosomal retention compartment (Tomas et al., 2010; Karleusa et al., 2018; see Lucin et al., 2015 for an in-depth review of cytomegalovirus endosomal trafficking in the context of immune evasion).

Several cellular systems are involved on the host side of the cVAC biogenesis equation. As an example, microtubules are involved in bending the nuclear membrane around the cVAC, resulting in the reniform shape characteristic of nuclei in HCMV infected cells (Buchkovich et al., 2010). The importance of microtubules is illustrated by the observation that disruption of microtubules with nocodazole leads to disruption/dispersal of the cVAC, which was able to reverse within 90 min after removal of the drug (Indran et al., 2010). Overexpression of the N-terminus of Bicaudal-D1 (a protein involved in secretory trafficking through interactions with dynein, Rab6, and the HCMV inner tegument protein pp150) also results in disruption of the cVAC and diminished infectious yields (Indran et al., 2010). In addition, BiP/GRP78 (an endoplasmic reticulum (ER) chaperone), dynamin-2 (important for glycoprotein biogenesis and transport), and syntaxin 5 (a SNARE family cellular trafficking regulator) are all required for cVAC formation and efficient production of infectious virions (Buchkovich et al., 2009, 2010; Indran et al., 2010; Archer et al., 2017; Cruz et al., 2017). In addition, phosphorylation of the Golgi peripheral membrane protein, Grasp65, is important for the Golgi remodeling that occurs during cVAC biogenesis, with the phosphorylation hypothesized to be associated with activation of mitotic kinases during HCMV infection (Rebmann et al., 2016). While the full extent of the interactions between viral and cellular factors remains to be mapped, it is clear that HCMV utilizes a complex multi-factorial program to modulate the host environment and promote replication.

We now turn to our main question: which pathway(s) does HCMV employ for acquisition of its secondary envelope and for transportation to the PM during release? As mentioned earlier, ultrastructural experiments indicated that HCMV envelopment and egress is via an early endosomal recycling compartment (Tooze et al., 1993). It was subsequently learned that purified virions, dense bodies, and exosomes produced by HCMV infected cells contain cellular proteins involved in vesicular trafficking, including markers of trans-Golgi network (TGN), early/recycling endosomes, and exosomal compartments (Varnum et al., 2004; Walker et al., 2009; Cepeda et al., 2010). This suggests that during envelopment and egress, the virus induces creation of a novel compartment and/or exploits vesicles involved in inter-organelle trafficking. Consistent with these hypotheses, transcripts for several proteins involved in membrane trafficking are upregulated during HCMV infection (Hertel and Mocarski, 2004), including Rab27A, a regulator of lysosome-related organelles (LRO) and exocytosis via SVs, and SNARE protein syntaxin 3 (STX3), which participates in membrane fusion during exocytosis. Additionally, secondary envelopment occurs via membranes containing Rab3, a key regulator of Ca^2+^-dependent SV exocytosis (Homman-Loudiyi et al., 2003; Schluter et al., 2006). The apparent importance of Rab27A was confirmed when it was shown to localize to vesicles throughout the center of the cVAC, purify with mature virions, and be required for the efficient production and release of nascent virions (Fraile-Ramos et al., 2010). The upregulation of STX3 was confirmed at the protein level, and the result was then extended by showing that knockdown of STX3 resulted in reduced infectious yields and reduced expression of lysosomal glycoproteins (Cepeda and Fraile-Ramos, 2011). Lastly, the lipid profile of HCMV virions produced from fibroblasts more closely mirrors that of synaptic vesicles, a type of SV, than the typical profile found within uninfected fibroblasts (Liu et al., 2011). Based on these and other observations, the weight of published evidence now suggests that HCMV envelopment and egress occur via a Rab3/Rab27A-linked LRO- or SV-related pathway, but this has not been proven and important questions remain.

While much has been learned, a comprehensive model of HCMV maturation has yet to be articulated. An important limitation is the dearth of relevant, highly integrated systems-level data connecting viral and cellular gene expression to protein localization, organelle function, and metabolic activity. To begin to address this part of the puzzle, we analyzed previously published, independently obtained, transcriptional and proteomic datasets in an attempt to predict the likely effects of HCMV infection on ERC-associated trafficking pathways (Gurczynski et al., 2014; Weekes et al., 2014). Our analysis suggests that relative to uninfected cells, HCMV-induced regulation of trafficking events through the ERC leads to a decrease in the net flux of exocytic pathways while the rate of endocytic transport remains relatively consistent. We also identified infection-associated changes in gene expression that suggest increased outward flow of SV traffic. The results of these relatively unbiased, systems-level analyses were remarkably consistent with numerous previously published observations, suggesting that the approach has merit.

The information and model presented here provide a framework for understanding virion envelopment and egress. This will provide a roadmap for future hypothesis-driven experimental examination of these essential processes, as well as for identification of regulatory elements that can be targeted to inhibit HCMV infection.

## Materials and Methods

### Viruses

Analyses reported here utilized data from published studies that employed HCMV strains AD169 (ATCC), Towne (Thomas Shenk; Princeton University, Princeton, NJ, United States), Merlin (Richard Stanton; Cardiff University, Cardiff, United Kingdom), and the AD169-derived variant pAD/Cre (Dong Yu; Washington University, St. Louis, MO, United States). All strains were grown in low-passage primary human foreskin fibroblasts (HFFs) using complete Dulbecco’s modified Eagle medium supplemented with 5–10% fetal bovine serum and viral titrations were done using standard plaque assays (Das et al., 2014; Gurczynski et al., 2014; Weekes et al., 2014).

### Datasets

Generation of the AD169 transcriptional microarray dataset in our laboratory was previously described (Gurczynski et al., 2014). Low passage HFFs were mock treated or infected at a high MOI equal to 3.0 using HCMV strain AD169 and incubated for 12 or 96 h. Upon harvest, RNA was Trizol extracted, and RNA quality was assessed using an Agilent Bioanalyzer. Only RNA with an integrity score of 8/10 or higher was accepted for downstream processes. RNA samples were then loaded onto Illumina HT-12 v4 human bead array chips for detection. Hybridization and reading of the bead arrays were done by the Advanced Genomics Technology Center (AGTC) at Wayne State University. Results were reported as individual signal intensities. Although not previously reported, we simultaneously collected a parallel transcriptional dataset for strain Towne, under the same conditions, and using the same experimental design. All bead array data are available from the Gene Expression Omnibus database (accession numbers: GSE50955 [Mock and AD169] and GSE112514 [Towne]).

The proteomic dataset was also described previously (Weekes et al., 2014). HFFs were infected at an MOI of 10.0 using the HCMV strain Merlin. At designated times after infection, PM and whole-cell lysate (WCL) samples were prepared, enzymatically digested, and labeled with 8-plex and 10-plex tandem mass tag reagents. Labeled samples were then subjected to liquid chromatography and tandem mass spectrometry. For our analyses, we used information from the PM2 (PM) and WCL2 (WCL) datasets, which were reported in terms of abundance relative to peak levels of detection.

### Computational Analyses

The transcriptional and proteomic datasets were analyzed using R (v3.4.1). Transcriptional data was initially filtered using the significance analysis of microarrays (SAM) function set to a false discovery rate of 0.01 within BRB-ArrayTools (v. 4.2.0 beta 2; Richard Simon and the BRB-ArrayTools Development Team). Proteomic data was filtered to include only host-specific genes. Data from samples harvested at 12 and 96 hpi were used to determine the log_2_ fold change of infected cells relative to mock infected cells (log_2_FC). The change in log_2_FC (Δlog_2_FC) was also calculated to summarize patterns in regulation of transcript or protein abundance throughout infection. The log_2_FC and Δlog_2_FC metrics were used to apply an additional biological significance filter to both datasets based on a log_2_FC < -1 or >1 for any time point (12 or 96 h) or an overall Δlog_2_FC value < -1 or >1. The resulting gene lists were used to assess overrepresentation of gene ontology (GO) terms within the datasets.

Gene ontology analysis was done by individually importing the filtered transcriptional and proteomic datasets into Cytoscape (v3.6.0) using the BiNGO package. Biological process annotation was done using updated ontology and annotation definitions downloaded from http://www.geneontology.org/; the definitions were validated on 26 September 2017 and 12 January 2018, respectively. Overrepresentation was determined using a Benjamini–Hochberg corrected hypergeometric test (corr. *p*-value; α = 0.05). In addition to network maps, lists of genes from each dataset annotated by the GO terms ‘Secretion,’ ‘Intracellular transport,’ and ‘Vesicle-mediated transport’ were exported for further use.

Next, the GO term-associated gene lists were used to select transcripts and proteins with potential roles in ERC trafficking from the respective datasets. The R package vennDiagram was used to illustrate the overlap of gene annotation for each GO term between the datasets. Scatterplots were generated using ggplot2 and ggpmisc to compare the abundance data within and between the datasets over time. Heatmaps were made using limma, gplots, and dendextend. Clustering was done through an average linkage method and a Pearson’s correlation coefficient-based distance matrix. Rows were individually normalized, and results were reported as *z*-scores based on the standard deviation around the mean log_2_ abundance for each row.

Determination of infection-induced impacts on trafficking pathways (**Figure 4G** and **Supplementary Table S1**) was done as follows: genes with significantly altered expression profiles over the course of infection and annotated by the GO terms “Secretion,” “Intracellular transport,” or “Vesicle-mediated transport” were compiled from both the protein and transcript datasets generating a list of 1,015 unique genes. Of these genes, proteins known to be key regulators of trafficking events during intracellular transport were selected resulting in a final list of 134 different genes to be used for further analysis. Due to lack of rate specific information, genes with Δlog_2_FC > 0 were given a value of 1 and genes with Δlog_2_FC < 0 were given a value of -1 to represent general upregulation or downregulation of gene expression, respectively. Then, all values for a given gene, across all sample sets (WCL, PM, AD169, and Towne), were averaged generating a single metric representing cumulative change in expression. Genes with changes in expression between -1 and 1 represent minor conflicts between datasets but majority consensus, while values of 0 represent major conflicts between the datasets. The list of 134 key regulators was then manually annotated and each gene was assigned a function in intracellular trafficking based on evidence from primary literature. Genes were also assigned a value of 1 if they positively regulated the annotated process, -1 if they were inhibitory, or 0 if the reported regulatory roles were inconclusive. Lastly, the change in expression metric was multiplied by the binary positive or negative regulatory metric resulting in an aggregate measure of the change in flux along particular pathways. These cumulative measures were then mapped onto the individual pathways detailed in **Figure 1** resulting in the trafficking schematic detailed in **Figure 4G**.

All remaining graphs were generated using ggplot2 and virdis for color scaling.

All datasets and code used for analysis are hosted at https://github.com/wclose/Close_HCMVEgress_FrontMicrobiol_2018.

### Transmission Electron Microscopy

Infection and imaging of cells infected with HCMV variant pAD/Cre was previously described by our lab (Das et al., 2014). In brief, HFFs were infected using a MOI of 0.3 and infections progressed until 120 hpi. Cells were fixed and shipped to Dr. Hong Yi at the Robert P. Apkarian Integrated Electron Microscopy Core Facility of Emory University (Atlanta, GA, United States) for staining and imaging.

## Results

### Curation of Systems-Level Datasets

**FIGURE 2:**
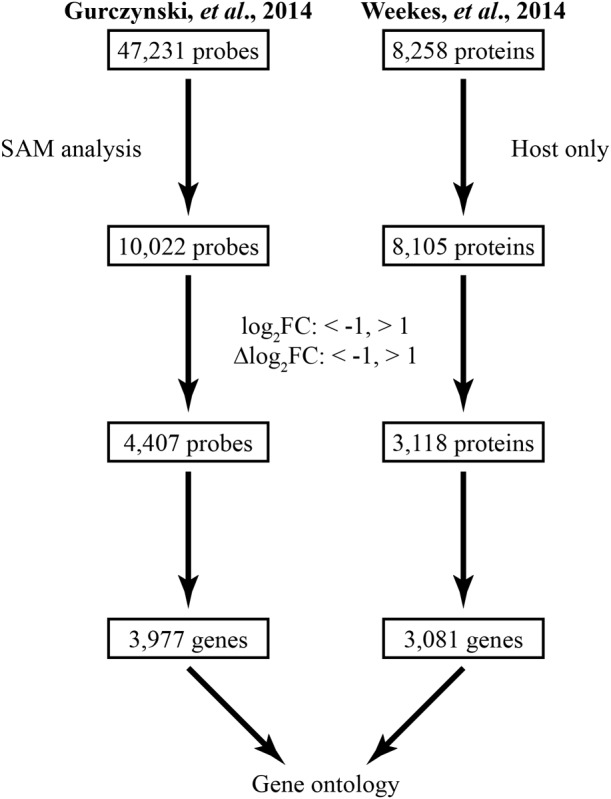
Gene curation scheme for the transcriptional and proteomic datasets. The transcriptional dataset **(left)** was curated using SAM analysis (false discovery rate = 0.01) before being subjected to biological significance thresholds based on the log_2_FC and Δlog_2_FC. The proteomic dataset **(right)** was similarly filtered. First, host cellular proteins were chosen, then, only proteins that passed the log_2_FC-, Δlog_2_FC-based significance thresholds were selected. The resulting lists of 3,977 and 3,081 unique genes curated from the transcriptional and proteomic datasets, respectively, were used for gene ontological analysis.

To generate a model of ERC trafficking within HCMV infected cells, we used two previously published datasets which analyzed the transcriptional and proteomic expression profiles of cellular genes at multiple time points throughout infection (Gurczynski et al., 2014; Weekes et al., 2014). Due to the large amount of information contained within each dataset, we used a series of significance-based filtration steps to focus our analysis (**Figure 2**).

The transcriptional dataset was obtained using arrays that included 47,231 individual mRNA-targeting probes mapping to over 28,000 host genes. The dataset contains expression data for fibroblasts infected with HCMV (AD169 or Towne; MOI 3.0), at early (12 hpi) and late (96 hpi) time points of infection, as well as for mock-infected cells. Because of our interest in changes to normal cellular processes, we performed a SAM analysis, which enables identification of genes whose transcript levels differ significantly relative to mock-infected cells under the assorted infection conditions. The resultant dataset contained 10,022 probes.

We then calculated the log_2_FC and the Δlog_2_FC for each probe. The Δlog_2_FC provides a single summary metric for changes in abundances over the course of infection:

$$\begin{array}{c} \Delta \log_{2}\mathrm{FC}\;\;=\;\;\log_{2}\left(\frac{96\;\mathrm{hpi}}{\mathrm{mock}}\right)-\;\log_{2}\left(\frac{12\;\mathrm{hpi}}{\mathrm{mock}}\right) \end{array}$$

Both the log_2_FC and Δlog_2_FC were used to refine the transcriptional dataset by only including probes that surpassed biological significance thresholds of log_2_FC < -1 or > 1 at any time point or an overall Δlog_2_FC < -1 or > 1. From the original dataset, 4,407 mRNA probes remained, mapping to 3,977 unique genes.

The proteomic data also underwent filtration. The initial dataset contained protein abundance data based on liquid chromatography coupled tandem mass spectrometry using HCMV Merlin infected fibroblasts (MOI 10.0) compared to mock-infected cells, at multiple time points, and divided into WCL and PM fractions. For our analysis, and to improve comparability between the datasets, we only used abundance data from the mock, 12 hpi, and 96 hpi samples. Of the initial 8,258 proteins in the dataset, we filtered out 153 viral proteins, leaving 8,105 host proteins (WCL = 7,491; PM = 2346) for further analysis. Data for these proteins was filtered through the same log_2_FC-, Δlog_2_FC-based threshold as employed for the transcriptional data. This resulted in a final list of 3,118 proteins representing 3,081 unique genes. Use of the Δlog_2_FC metric enabled comparisons between transcriptional data, quantified based on signal magnitude, and proteomics data, expressed in terms of percent peak abundance over time.

### HCMV Infection Leads to Changes in Transcript and Protein Abundances That Connect to Cellular Transport Systems

Following the fold-change and significance-based filtration of the transcriptional and proteomic datasets, we sought to identify the cellular processes most affected during HCMV infection, with an emphasis on ERC-related systems. To achieve this, we employed GO classification of biological processes to annotate the gene lists generated from each dataset and look for trends in targets of regulatory modifications.

**FIGURE 3:**
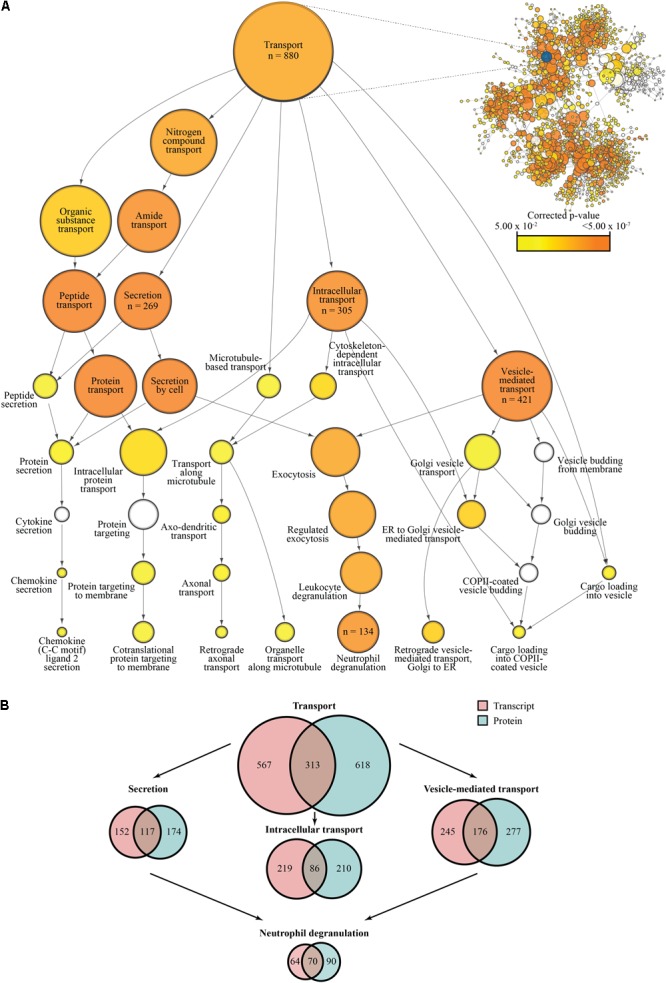
Gene ontology-based analysis of transcriptional and proteomic regulation during HCMV infection. **(A)** Network map of overrepresented GO terms following significance filtration of the transcriptional dataset. The “Transport” node and its descendant nodes have been enlarged to better visualize relevant processes. Descendant nodes are terms nested within a given parent node; relationships between parent nodes and descendant nodes are indicated by directional arrows representing the shift from general to more specific classifiers. Genes are classified based on the deepest level of annotation available and node areas are proportional to the number of genes classified by each term with some genes being classified by more than one term at a given level in the hierarchy; specific values have been listed for nodes of interest. Reported *p*-values are Benjamini–Hochberg corrected (α = 0.05); uncolored nodes were not significantly overrepresented. A comparable network map based on overrepresentation of GO annotations in the filtered proteomics dataset may be found in **Supplementary Figure S1**. **(B)** Comparison of gene annotation between the transcriptional and proteomic datasets. Values represent the number of overrepresented genes per term.

Of the 3977 genes identified in the transcriptional dataset, 3154 (79.3%) mapped to at least one GO term. Of the 3081 genes identified from the proteomic dataset, 2,892 (93.9%) were also successfully mapped. Based on overrepresentation of GO terms, network maps were created highlighting the impact of HCMV induced changes in global regulatory schemes at the transcriptional and proteomic levels (insets, **Figure 3A** and **Supplementary Figure S1**, respectively).

Due to our interest in cytoplasmic trafficking events and how they may relate to HCMV egress, we focused our analysis on the GO term “Transport” and its descendant GO terms (**Figure 3A** and **Supplementary Figure S1**). Of the successfully annotated genes, 880 (27.9%; corr. *p*-value = 1.5237 × 10^-5^) and 931 (32.2%; corr. *p*-value = 3.1082 × 10^-25^) mapped to “Transport” from the transcriptional and proteomic datasets, respectively, representing significant degrees of enrichment. Regarding the descendant nodes, we concentrated our analysis on “Secretion,” “Intracellular transport,” and “Vesicle-mediated transport” as they appeared most relevant to the process of HCMV egress. Of the annotated transcript-associated genes, 269 (8.5%; corr. *p*-value = 3.4799 × 10^-8^) mapped to “Secretion,” 305 (9.7%; corr. *p*-value = 1.1582 × 10^-6^) mapped to “Intracellular transport,” and 421 (13.3%; corr. *p*-value = 9.9166 × 10^-9^) mapped to “Vesicle-mediated transport.” Of the successfully mapped protein-associated genes, “Secretion” represented 291 (10.0%; corr. *p*-value = 6.8507 × 10^-18^), “Intracellular transport” accounted for 296 (10.2%; corr. *p*-value = 3.8953 × 10^-11^), and “Vesicle-mediated transport” captured 453 (15.7%; corr. *p*-value = 1.7845 × 10^-24^). Interestingly, despite both datasets being generated from fibroblasts, “Secretion” and “Vesicle-mediated transport” both shared a sequence of significantly enriched descendant nodes related to exocytosis, ending with “Neutrophil degranulation.” Neutrophil degranulation is a process that involves Ca^2+^-dependent cytoskeletal remodeling and targeted release of SV-like cargo required for immunological responses (reviewed in Lacy, 2006). The “Neutrophil degranulation” GO term includes 134 of the transcript-associated genes, such as LAMP1/2, NFKB1, CD44, STXBP2, and SNAP25, (4.2%; corr. *p*-value = 1.9979 × 10^-6^) and 160 of the protein-associated genes, including LAMP1/2, RAB3A/D, STXBP2, CEACAM1, PECAM1, (5.5%, corr. *p*-value = 2.6240 × 10^-17^), thus explaining a substantial proportion of the enrichment seen with the associated ancestor nodes.

While the transcriptional and proteomic datasets had roughly equivalent numbers of genes map to each term and identified very similar sets of processes (**Figure 3A** and **Supplementary Figure S1**), only 28–52% of the annotated genes were similar across both datasets (**Figure 3B**). The differences in representation within these groups can likely be ascribed to these processes being modulated at the transcriptional and protein levels through mechanisms such as mRNA and protein stability, translation efficiency, and post-translational modifications.

### HCMV Infection Induces Coordinated Changes in the Abundance of ERC Regulators

**FIGURE 4:**
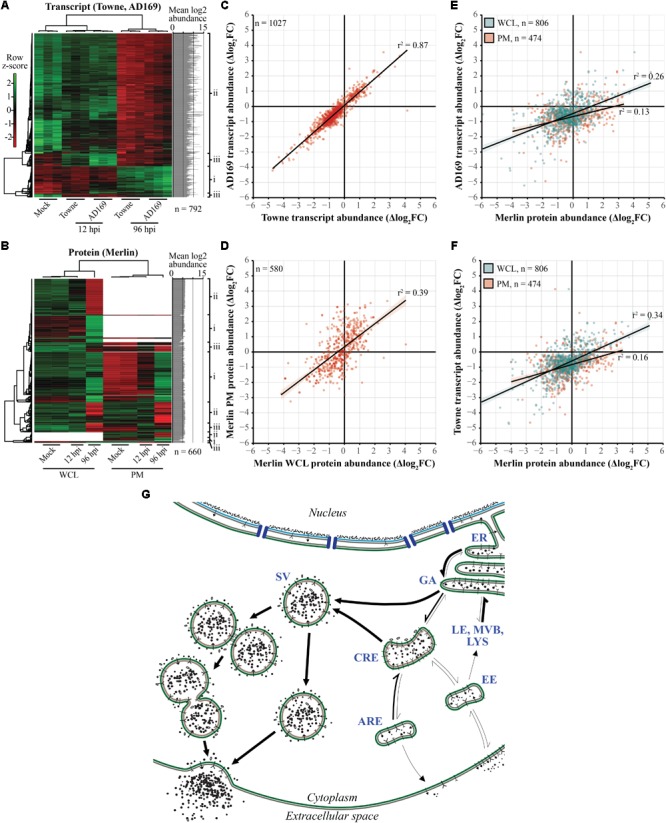
Expression profiles of cellular genes required for transport are modulated as a function of time during HCMV infection. **(A,B)** Heatmaps of transcript **(A)** and protein **(B)** abundances demonstrating fluctuations over the course of infection. Data includes genes annotated by the “Secretion,” “Intracellular transport,” or “Vesicle-mediated transport” GO terms. Clustering was done using an average linkage method and Pearson’s correlation coefficient-based distance matrix resulting in the displayed row and column dendrograms. Rows are individually normalized based on standard deviation around the mean log_2_ abundances (*z*-score; graphs at right-hand side). Right-hand side labels indicate genes that are (i) upregulated over the course of infection, (ii) downregulated over the course of infection, or (iii) up- or downregulated early in infection but return to mock-like levels late in infection. **(B)** Regions without color indicate missing data. **(C–F)** HFF cellular expression patterns of genes annotated by aforementioned GO terms were summarized (Δlog_2_FC) and plotted relative to each other to determine correlation of regulatory mechanisms induced by infection. **(C)** Comparison of relative transcript levels in Towne and AD169 infected cells (MOI 3.0). **(D)** Comparison of relative protein abundances between WCL and PM fractions of Merlin infected cells (MOI 10.0). **(E)** Comparison of Merlin protein levels from WCL and PM fractions against transcript levels of AD169 infected cells. **(F)** Comparison of WCL and PM protein fractions from Merlin infected cells versus transcript levels in Towne infected cells. **(G)** HCMV induced shifts in net flux of ERC trafficking behaviors based on patterns in Δlog_2_FC based abundances and literature-based roles in transport regulation. Thicker arrows indicate the predicted flow of traffic relative to uninfected cells (see **Supplementary Table S1** for more information). Apical recycling endosomes (ARE), common recycling endosomes (CRE), early endosomes (EE), endoplasmic reticulum (ER), Golgi apparatus (GA), late endosomes (LE), lysosomes (LYS), multivesicular bodies (MVB), secretory vesicles (SV).

Based on the GO analysis, we looked closer at the relationships between the transcriptional and proteomic datasets (**Figure 4**). We extracted the gene lists associated with the “Secretion,” “Intracellular transport,” and “Vesicle-mediated transport” GO terms for both datasets, using the combined list to further filter the pool of genes. Because we were interested in comparing patterns in regulation between the two datasets, we removed any genes represented in only one of the original datasets. The final transcriptional dataset included 1,027 probes for both AD169- and Towne- infected samples. The final proteomic dataset included 1,057 proteins in the WCL fraction and 631 proteins in the PM fraction.

Because virion envelopment and egress occur late in infection, we employed heatmaps to display changes in the abundance of gene products from the transcriptional and proteomic datasets as a function of time after infection (**Figures 4A,B**). Within each row, data for individual genes were normalized around the mean log_2_ abundance and clustering was done based on the similarity of expression patterns. Regions within the protein-based heatmap clustered first on the completeness of the data, then on patterns of abundance. Within each dataset, three distinct regulatory profiles are apparent relative to mock: (i) upregulation over the course of infection, (ii) downregulation over the course of infection, and (iii) up- or downregulation early in infection that returns to mock-like levels late in infection (right-hand side labels, **Figures 4A,B**). These regulatory patterns occurred across a wide range of abundances in the transcriptional data, a feature which was obscured in the proteomic data as a result of the format used to report values in the raw dataset, thus the protein-associated plot shows very little variation (right-hand side graphs, **Figures 4A,B**).

To better understand the impact on host trafficking processes, we used scatter plots to compare the expression profiles using the Δlog_2_FC summary statistic. The datasets were initially compared within themselves via linear regression to establish a baseline for comparison. For the transcriptional dataset, we compared the AD169 and Towne subsets to each other (**Figure 4C**), whereas the WCL and PM fractions were compared in the protein dataset (**Figure 4D**). As expected, subsets within the transcriptional data had a higher degree of correlation (*r*^2^ = 0.87) compared to those of the protein data (*r*^2^ = 0.39). As might be expected, the transcriptional data correlated more with data from the WCL fraction than the PM fraction (**Figures 4E,F**). Overall, the observed patterns demonstrate the multi-faceted and coherent effect HCMV infection has on shaping the expression of host genes involved in cytoplasmic trafficking.

Finally, the observed changes in relative gene expression were mapped onto pathways connecting the major components of the ERC (**Figure 4G**). Shifts in relative flux along trafficking routes were predicted based on the mean Δlog_2_FC across all datasets and whether gene products are known positive or negative regulators (**Supplementary Table S1**). As an example, a decrease in the abundance (Δlog_2_FC < 0) of a negative regulator of endocytosis potentially indicates less inhibition and therefore a net increase in flux along that pathway. In the absence of quantitative rate-specific data for transport through the ERC, the magnitude of impact for each change in gene expression was assumed to be equivalent. Consistent with prior observations of cargo sequestration near the center of the cVAC during infection (Hook et al., 2014), ERC exocytic pathways associated with early or recycling endosomes tended to be negatively impacted. Conversely, other pathways, such as the SV-associated exocytosis, appeared to be favored during infection suggesting a potential mechanism of virion egress.

### HCMV Induces Changes in ERC Regulators Suggesting Increased Flux Through the SV Pathway

**FIGURE 5:**
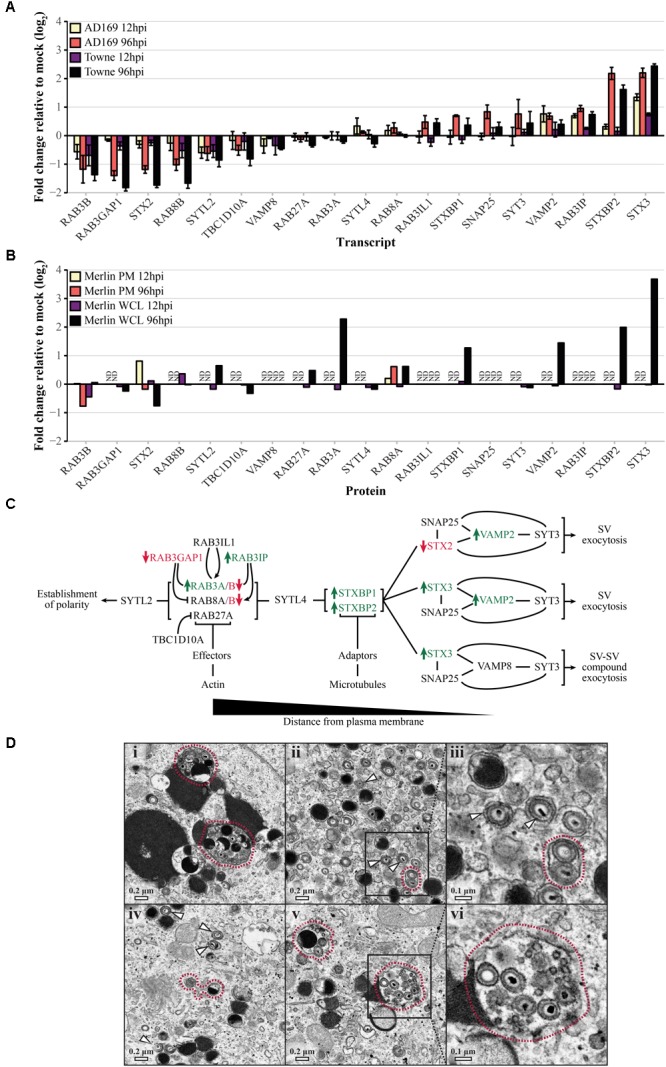
Human cytomegalovirus (HCMV) induced changes to transport-associated pathways in HFFs favor SV exocytosis relative to mock infected cells. **(A)** Relative transcript levels of genes required for SV exocytosis during infection with HCMV AD169 or Towne (MOI 3.0) at early (12 hpi) and late (96 hpi) time points. **(B)** Relative protein abundances in WCL or PM fractions from HCMV Merlin-infected cells (MOI 10.0) at early and late time points. Samples for which protein data was unavailable are marked as not determined (ND). **(C)** Schematic outlining trafficking events required for SV exocytosis. As vesicular traffic proceeds toward the right-hand side of the diagram, SVs contact the plasma membrane and undergo Ca^2+^-dependent individual or compound exocytic events. Genes which are significantly upregulated (log_2_FC > 1; green) or downregulated (log_2_FC < –1; red) at any time point based on the combined transcriptional and proteomic data are indicated. **(D)** Representative images of HFFs infected by the AD169-derived strain, pAD/Cre (MOI 0.3), demonstrating the accumulation of individual (white arrows) and compound (red outlines) vesicles containing mature viral particles. Images in panels (iii) and (vi) show an enlarged view of the areas indicated in panels (ii) and (v), respectively.

Due to the accumulated evidence that SV-associated pathways are involved in virion egress, we compared the relative abundances of transcripts (**Figure 5A**) and proteins (**Figure 5B**) for genes known to regulate SV exocytosis (**Figure 5C**). Because many of the events related to SV docking and fusion at the cell surface are mediated through low abundance, membrane-bound proteins, sensitivity limitations prevented several from being detected in the PM fraction of the proteomic dataset. In contrast to the apparent decrease in exocytic, but not endocytic, transport via early and recycling endosomes (**Figure 4G**), expression of SV exocytosis regulatory genes shifted in a direction indicative of upregulation of the pathway as a whole (**Supplementary Table S1** and **Figure 5C**). Consistent with previously published results (Cepeda and Fraile-Ramos, 2011), not only did levels of positive regulators (RAB3A, RAB3IP, STXBP1/2, STX3, VAMP2; log_2_FC > 1; green) increase over the course of infection, levels of multiple negative regulators decreased over the same time frame (RAB3GAP1, RAB8B, RAB3B; log_2_FC < -1; red). Interestingly, the data suggest elevated activity for both individual- and compound-exocytic pathways.

To assess the potential relevance of these observations, we examined a collection of electron micrographs previously obtained by our laboratory (Das et al., 2014) (**Figure 5D**). In images of cells infected with the HCMV AD169-derived strain, pAD/Cre (MOI 0.3), we were able to identify individual (white arrows) and compound (red outlines) vesicles in the cytoplasmic space containing mature viral particles. Of 89 enveloped virions, 68.5% were inside vesicles occupied by only one virion (as visualized in two dimensions), whereas the remaining 31.5% were present in shared vesicles. While it is unclear whether single- and multiple-virion vesicles are capable of delivering infectious payload to the PM, the quantity of each and the apparent integrity of the contained virions suggest that both single and compound vesicles may play a role in HCMV virion egress.

## Discussion

**FIGURE 6:**
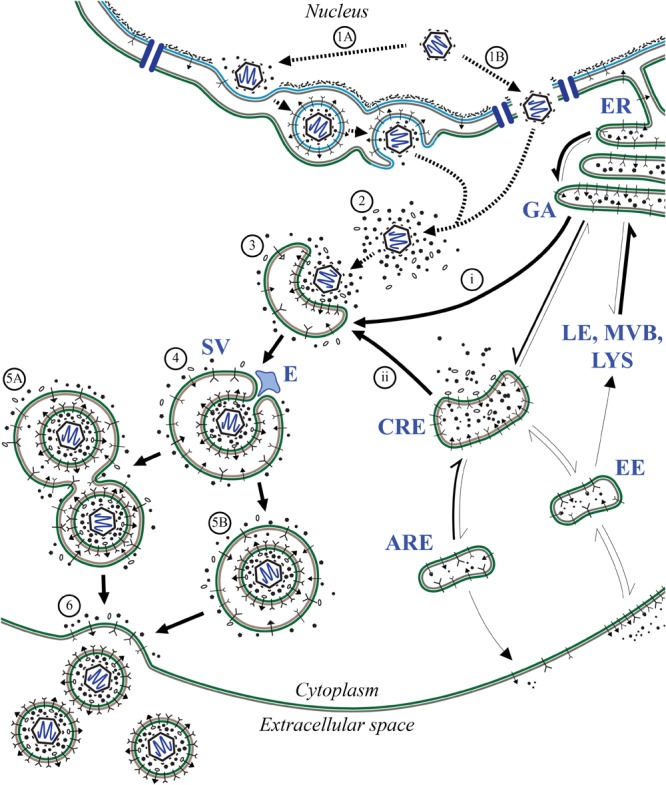
Model of HCMV maturation and egress through the ERC. Following analysis of regulatory patterns involved in ERC trafficking (**Figures 2–4**), key aspects of the viral replication cycle were mapped to affected pathways to predict changes in ERC pathways relevant to HCMV assembly and egress. Following genome replication and packaging into fully assembled capsids, capsids are transported out of the nucleus of infected cells. Nuclear egress occurs via primary envelopment at the inner nuclear membrane and subsequent de-envelopment at the outer nuclear membrane **(1A)** or by passage through virally induced structural aberrations in the nuclear membrane (Buchkovich et al., 2010; Klupp et al., 2011) **(1B)**. Once in the cytoplasm, capsids acquire the proteinaceous tegument layer **(2)** which serves as a scaffold for interactions with enveloping membranes **(3)**. Membranes used for secondary envelopment are derived from ER/GA **(i)** and endosomal **(ii)** compartments. During secondary envelopment, components of ESCRT machinery are used for scission of budding virions **(4)** resulting in fully matured virions contained within compound **(5A)** and individual **(5B)** SV-like vesicles. These transport vesicles are translocated to the PM where the outer bilayer fuses **(6)** thus releasing nascent mature virions for infection of neighboring cells. Dashed arrows denote findings from previous studies; solid arrows denote observations from this work. Thickness of solid arrows indicates relative change in net flux compared to uninfected cells with thicker arrows being the favored direction of transport. Apical recycling endosomes (ARE), common recycling endosomes (CRE), endosomal sorting complex required for transport (ESCRT; E), early endosomes (EE), endoplasmic reticulum (ER), Golgi apparatus (GA), late endosomes (LE), lysosomes (LYS), multivesicular bodies (MVB), secretory vesicles (SV).

Despite the public health burden imposed by HCMV, there is a deficiency in the number and target diversity of licensed treatment options. A major factor contributing to these inadequacies is the lack of information regarding HCMV maturation, particularly during cytoplasmic stages of the viral replication cycle. As shown for human immunodeficiency virus 1, successful control of lytic viral infections is best accomplished through simultaneous targeting of multiple critical control points throughout virus replication. In addition to suppressing viral spread, this also reduces the likelihood of developing resistance to antivirals. Because HCMV replicates in cells that express thousands of genes, there is a vast virus–host interface that has only begun to be explored and only a fraction of potentially druggable targets have been identified. In this work, we investigated the final stages of HCMV virion maturation by analyzing infection-induced regulatory schemes used to alter cellular transport through the ERC. By utilizing transcriptional and proteomics datasets to provide new information about the effects of HCMV infection on the host ERC, we assembled a model for HCMV envelopment and egress that builds on over 40 years of published research (**Figure 6**).

Through use of GO annotation (**Figure 3**) and analysis of relative gene expression patterns over time (**Figures 4, 5**), our results reinforce and extend the contemporary model of HCMV maturation. Our model predicts that changes in the flux of ERC pathways lead to accumulation of transport machinery and associated cargo within discrete regions distributed throughout the cVAC. This suggests a possible mechanism for the sequestration of immune signaling molecules as seen by others (Hook et al., 2014), and may also facilitate virion assembly by concentrating and ordering virion structural components within areas of assembly at the interface of Golgi-derived and endosomally-derived compartments within the cVAC (Tooze et al., 1993; Cepeda et al., 2010; Das and Pellett, 2011; Schauflinger et al., 2013).

Based on the observed patterns and the requirement to maintain cellular homeostasis during the protracted HCMV replication cycle, we predict that this shift in the balance between endocytosis and exocytosis is offset by a corresponding increase in the net flux through SV-like pathways. This supports the current hypothesis that HCMV envelopment and egress occur via a Rab3A/Rab27A-linked LRO- or SV-like pathway. To this end, the model is consistent with previous observations that several SV trafficking proteins are associated with mature virions (Homman-Loudiyi et al., 2003; Varnum et al., 2004; Cepeda et al., 2010; Fraile-Ramos et al., 2010; Cepeda and Fraile-Ramos, 2011), as well as with previous observations relating virion envelope lipid composition to that of synaptic vesicles (Liu et al., 2011), which rely on the highly conserved SV transport machinery (McMahon and Sudhof, 1995; Schiavo et al., 1997; Novick et al., 2006; Handley et al., 2007). In addition, SV exocytosis is mediated through a Ca^2+^ dependent mechanism and Ca^2+^ levels are substantially elevated throughout HCMV infection (Sharon-Friling et al., 2006). Lastly, genes involved in neutrophil degranulation were overrepresented in our analyses, a process which also relies on LRO/SV-related mechanisms (Lacy, 2006). Due to the highly conserved nature of these pathways across diverse cell types, our model encompasses mechanisms that may contribute to the wide cell tropism of HCMV.

Importantly, there are several limitations in our analysis:

(i) Although the datasets were positively correlated (**Figure 4**), the relatively low correlations between transcript and protein abundances are likely symptomatic of both methodological (e.g., differences in virus strain, MOI, method of detection) and biological (e.g., mRNA stability, post-translational modifications) confounding factors. It would be interesting to perform similar comparisons using the additional Merlin transcriptional and proteomic datasets from Tirosh et al. (2015) which became available well after we had begun the current analysis.
(ii) Linking expression changes to pathway flux will require experimental verification.
(iii) Current systems-level data related to HCMV is insufficient to robustly address readily visible questions.
(iv) We encountered several shortcomings with publicly available annotation databases related to intracellular or secretory trafficking systems. These included: a lack of supporting information regarding roles in trafficking events, the information contained in the databases is significantly behind current literature, and nomenclature differed depending on the field of study (e.g., immune cell degranulation versus endocrinology).

The agreement between our results and their consistency with prior observations in spite of these limitations, suggests a deep biological imperative. As is being done for other microbes, coordinated analyses of transcripts, proteins, protein modifications, and metabolic outcomes are needed. Such studies need to be conducted with multiple virus strains, in a variety of biologically relevant cell types, over a range of MOIs, and over a broad time course post-infection. In addition, ongoing investment is needed in updating and maintaining publicly accessible databases of host gene functions. Through studying the virus–host interactions occurring during the cytoplasmic stages of HCMV replication, we will also glean new knowledge of host cell biology. It will ultimately become important to distinguish cellular systems that behave differently in the context of infection from systems that function in an unperturbed manner.

The integrated roadmap presented here provides a foundation for interpreting experiments, past and present. It will prove useful for guiding future hypothesis-based experimentation as we work toward developing viable therapeutic alternatives and decreasing the public health burden of HCMV.

## Author Contributions

WC, JG, SG, and PP planned the analyses, which were conducted by WC, JG, and SG. PP collected the electron microscopic images with the help of Dr. Hong Yi of the Robert P. Apkarian Integrated Microscopy Core of Emory University. WC, JG, and PP collaboratively created the figures. WC drafted the text, with subsequent substantive contributions from JG, SG, and PP.

## Conflict of Interest Statement

The authors declare that the research was conducted in the absence of any commercial or financial relationships that could be construed as a potential conflict of interest.

## Abbreviations

Δlog_2_FC: change in log_2_FC over time
ARE: apical recycling endosomes
BiNGO: Biological Networks Gene Ontology
corr. *p*-value: Benjamini–Hochberg corrected *p*-value
CRE: common recycling endosomes
cVAC: cytoplasmic virion assembly complex
EE: early endosomes
ER: endoplasmic reticulum
ERC: endocytic recycling compartment
ESCRT; E: endosomal sorting complex required for transport
GA: Golgi apparatus
GO: gene ontology
HCMV: human cytomegalovirus
HFF: human foreskin fibroblast
Hpi: hours post infection
LE: late endosomes
log_2_FC: log_2_ fold-change relative to mock
LRO: lysosome-related organelle
LYS: lysosomes
MOI: multiplicity of infection
MVB: multivesicular bodies
ND: not determined
PM: plasma membrane
SAM: significance analysis of microarray
SV: secretory vesicle
WCL: whole-cell lysate
