# Supplementary material for: Infection-Induced Changes Within the Endocytic Recycling Compartment Suggest a Roadmap of Human Cytomegalovirus Egress
Source: Front Microbiol. 2018 Aug 22;9:1888. doi: 10.3389/fmicb.2018.01888 (PMC6113367; doi:10.3389/fmicb.2018.01888)
Supplement: Supplementary file 2 [file Image_1.PDF]

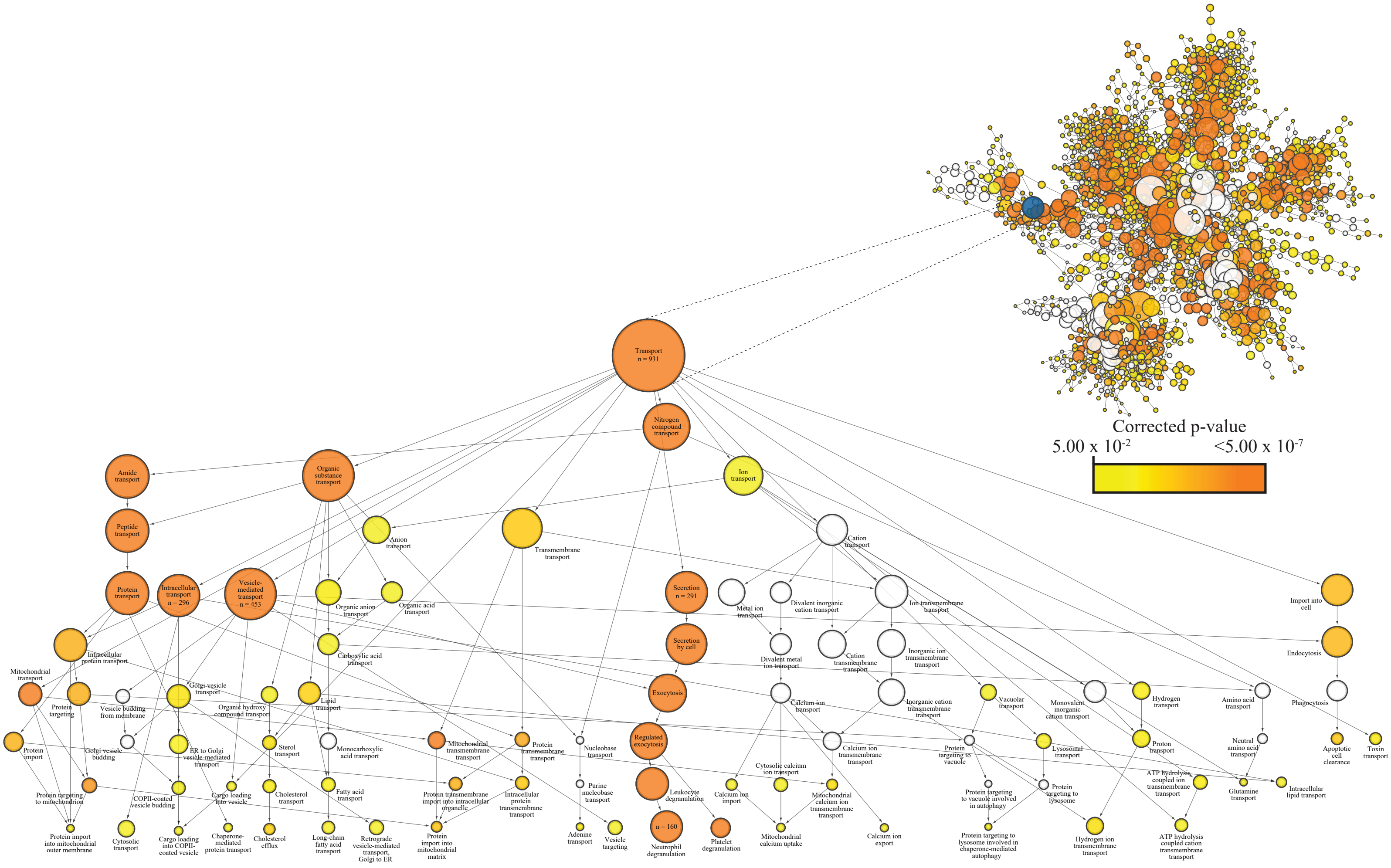

Figure S1. Network map of overrepresented gene ontology terms following significance filtration of the proteomic dataset. The "Transport" node and its daughter nodes have been enlarged to better visualize relevant processes. Node areas are proportional to the number of genes classified by each term; specific values have been listed for nodes of interest. Reported p-values are Benjamini-Hochberg corrected (α = 0.05); uncolored nodes were not significantly overrepresented.
